# Common Exposure to STL Polyomavirus During Childhood

**DOI:** 10.3201/eid2009.140561

**Published:** 2014-09

**Authors:** Efrem S. Lim, Natalie M. Meinerz, Blake Primi, David Wang, Robert L. Garcea

**Affiliations:** Washington University, St. Louis, Missouri, USA (E.S. Lim, D. Wang);; University of Colorado, Boulder, Colorado, USA (N.M. Meinerz, B. Primi, R.L. Garcea)

**Keywords:** Human polyomavirus, seroepidemiology, immunodeficiency diseases, viruses, children

## Abstract

STL polyomavirus (STLPyV) was recently identified in human specimens. To determine seropositivity for STLPyV, we developed an ELISA and screened patient samples from 2 US cities (Denver, Colorado [500]; St. Louis, Missouri [419]). Overall seropositivity was 68%–70%. The age-stratified data suggest that STLPyV infection is widespread and commonly acquired during childhood.

Polyomaviruses are nonenveloped double-stranded circular DNA viruses that infect a wide range of hosts, including humans. The capsid of the virus comprises primarily 72 pentamers of the major coat protein, VP1. Human polyomaviruses have been associated with several diseases (*1*). BK polyomavirus (BKPyV) has been associated with nephropathy in renal transplant recipients and JC polyomavirus (JCPyV) with progressive multifocal leukoencephalopathy in immunocompromised persons (*2*,*3*). Trichodysplasia spinulosa–associated polyomavirus (TSPyV) infection is linked to a rare skin disease in immunocompromised patients called trichodysplasia spinulosa (*4*). Furthermore, infection with Merkel cell polyomavirus (MCPyV) in rare instances leads to Merkel cell carcinomas, an aggressive form of skin cancer (*5*). Other polyomaviruses, including WU polyomavirus (WUPyV), KI polyomavirus (KIPyV), human polyomavirus 6, human polyomavirus 7, human polyomavirus 9, MW polyomavirus (MWPyV), STL polyomavirus (STLPyV), and human polyomavirus 12, have been identified in specimens from humans, but their role in disease remains to be defined (*1*).

Seroepidemiology has played an important role in establishing the link between human polyomaviruses and disease and in understanding infection dynamics. The seroprevalences of BKPyV and JCPyV range from 70% to 90% and 40% to 60%, respectively, with an age profile indicating high frequency of early-age infections and lifelong persistence (*6*–*8*). Seropositivity for MCPyV in healthy persons ranges from 25% to 64%; all patients with Merkel cell carcinoma are seropositive (*6*,*9*).

STLPyV was recently identified from fecal specimens from a child in Malawi (*10*). Viral DNA also was detected in fecal specimens from the United States and The Gambia, and STLPyV has been found in a surface-sanitized skin wart surgically removed from the buttocks of a patient with a primary immunodeficiency called WHIM (warts, hypogammaglobulinemia, infections, and myelokathexis) syndrome (*11*). These observations suggest that STLPyV might infect humans. We defined the seropositivity rate of STLPyV in humans using serum from 2 independent US sites (Denver, Colorado, and St. Louis, Missouri).

## The Study

**Figure 1:**
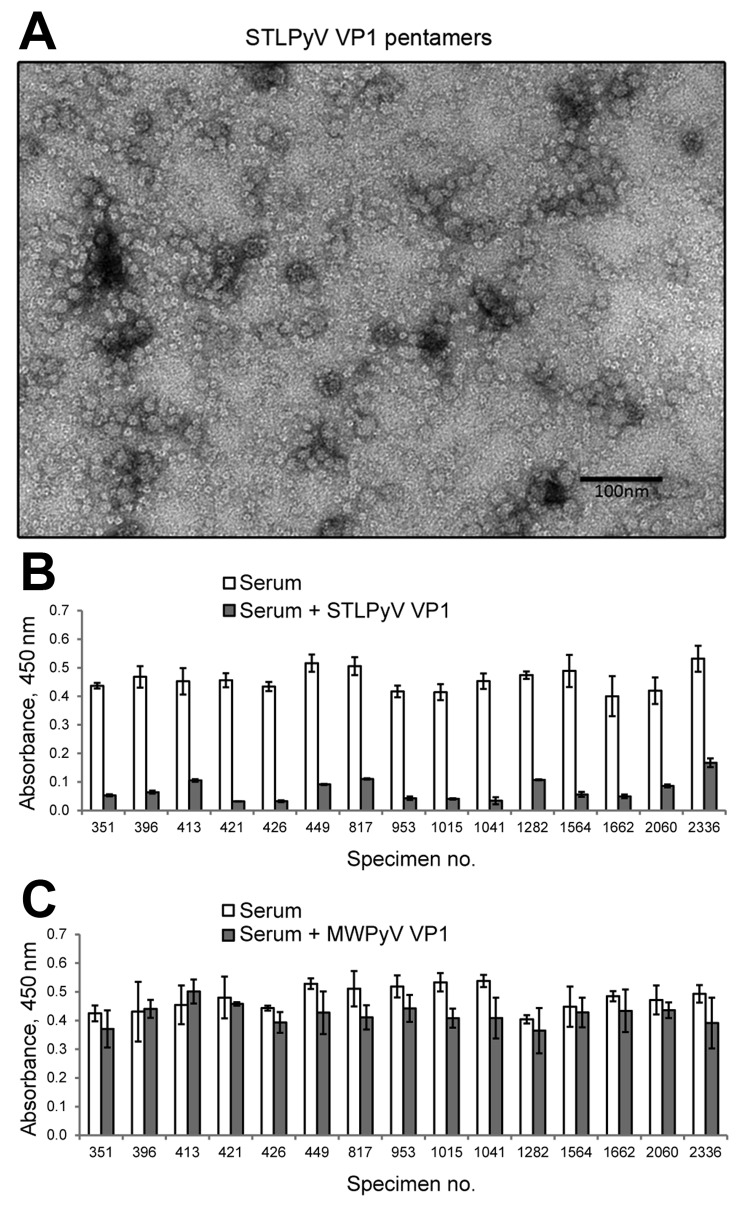
STL polyomavirus (STLPyV) ELISA. A) Electron microscopy image shows purified STLPyV VP1 capsomeres. Scale bar = 100 nm. B) Serum samples were pre-incubated in the absence (white bars) or presence of soluble STLPyV VP1 pentamers (gray bars), followed by the STLPyV-capture ELISA. Serum was tested in triplicate, and average absorbance values are shown. Error bars indicate SD. Representative data are shown. C) Seroreactivity to STLPyV in the absence (white bars) or presence of competition with MW polyomavirus (MWPyV) VP1 pentamers (gray bars) are shown. Serum was tested in triplicate, and average absorbance values are shown. Error bars indicate SD. Representative data are shown.

To determine the seropositivity for STLPyV, we developed a capture ELISA using recombinant glutathione S-transferase–tagged STLPyV VP1 capsomeres (Technical Appendix). Electron microscopy of the STLPyV capsomeres showed 10-nm pentamers characteristic of polyomaviruses (Figure 1, panel A). We assessed the specificity of the STLPyV ELISA by pre-incubating 24 serum samples in the presence and absence of soluble STLPyV VP1 pentamers before addition to the immobilized STLPyV glutathione S-transferase–tagged VP1. The ELISA signal intensity was markedly reduced when serum was pre-incubated with STLPyV VP1 pentamers (Figure 1, panel B; compare white bars to gray bars). This result indicates the ELISA seroreactivity could be self-competed with soluble STLPyV pentamers. STLPyV shares 55% aa identity in the VP1 region with its next most closely related polyomavirus, MWPyV (*10*). Therefore, we examined whether cross-reactivity existed between STLPyV and MWPyV VP1 capsomeres. Competition assays with soluble MWPyV VP1 pentamers showed limited interference with the ELISA seroreactivity (Figure 1, panel C; compare white bars to gray bars). This result indicates that there was no significant cross-reactivity between STLPyV and MWPyV VP1 capsomeres. Taken together, these data demonstrate that the ELISA was specific to STLPyV VP1.

**Figure 2:**
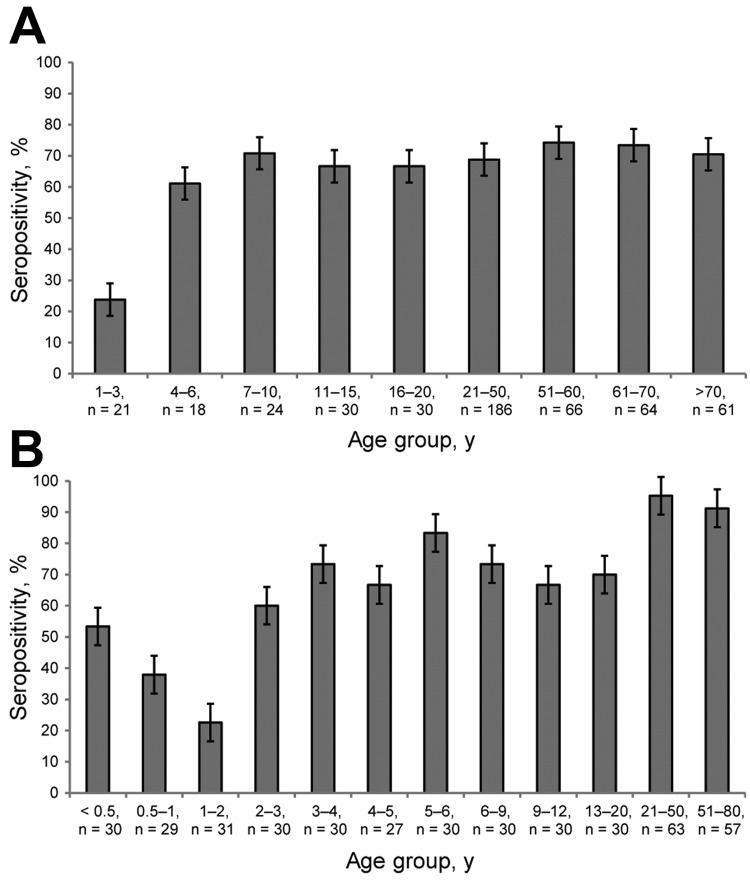
Age-specific seropositivity for STL polyomavirus (STLPyV) from serum specimens collected in Denver, Colorado, USA (A), and St. Louis, Missouri, USA (B). A total of 500 serum specimens from Denver and 417 from St. Louis were tested for seroreactivity to STLPyV VP1 proteins. Overall seropositivity in Denver was 68% and in St. Louis, 70%. Error bars indicate SD.

We screened 500 serum specimens collected from children and adults in Denver for antibodies against STLPyV. The overall seropositivity for STLPyV was 68.0% (Figure 2, panel A). Children 1–3 years of age had the lowest seropositivity rate (23.8%). In contrast, seroprevalence of children 4–20 years of age ranged from 61.1% to 70.8%. Similar seropositivity rates persisted in adults (>21 years of age), ranging from 68.8% to 74.2%.

We next examined a panel of 419 serum specimens from St. Louis that had a higher resolution of age-stratification in young infants. The overall seropositivity for STLPyV was 70.0% (Figure 2, panel B), similar to that in the specimens from Denver (68.0%). Seropositivity for STLPyV in infants dropped from 53.3% in the <0.5-year age group to 37.9% in the 0.5–1-year age group, with the lowest seropositivity of 22.6% in the 1–2-year group. In contrast, seropositivity rates for children >2 years of age were higher, ranging from 60.0% to 85.3%. Finally, seropositivity in adults (>21 years of age) ranged from 91.2% to 95.2%. Thus, these data indicate that exposure to STLPyV occurs during early childhood and that immune responses to STLPyV are maintained in adults.

## Conclusions

In our analysis of the seroepidemiology of STLPyV in 2 areas of the United States, we found that prevalence of the virus was similar (68.0%–70.0%). This prevalence is slightly higher than the 41.8% for MWPyV, the polyomavirus most closely related to STLPyV (*12*). However, the seropositivity of STLPyV is comparable to other human polyomaviruses (>60%), such as BKPyV, KIPyV, WUPyV, MCPyV, human polyomavirus 6, and TSPyV (*6*,*8*). We found no cross-reactivity with MWPyV VP1, the most closely-related polyomavirus that shares 55% aa identity in the VP1 region. Thus, the seroepidemiology strongly supports the notion that STLPyV is a bona fide infectious agent of humans.

Age stratification of the seropositive specimens suggested an initial waning of immune response followed by rapid seroconversion during childhood. In the St. Louis specimens, seropositivity was higher for the <0.5-year and 0.5–1-year age groups (53.3% and 37.9%, respectively) than for the 1–2-year group (22.6%). This observation was followed by an increase in STLPyV seropositivity in the 2–3-year group and older age groups (60.0%). Specimens from Denver were too few to reliably stratify the data to the same resolution. Nonetheless, we observed a similar trend in specimens from Denver where the seropositivity for the 1–3-year age group (23.8%) was lower than for the 4–6-year group (61.1%). These data indicate that immune responses to STLPyV decreased in the first 2 years of age, which suggests waning maternal antibodies. However, seropositivity was rapidly acquired thereafter, indicating high exposure of STLPyV in children. Because the specimens were selected from hospital-associated blood draws of children of unknown health status, seropositivity rates for healthy children might have varied somewhat from the results we obtained. Nonetheless, the trends we observed with STLPyV are similar to profiles that have been reported for JCPyV, BKPyV, TSPyV, MCPyV, WUPyV, and KIPyV (*6*,*13*–*15*).

STLPyV has not been clearly associated with any disease. However, the high overall seropositivity rate suggests widespread infection in the population at large. None of the pathogenic human polyomaviruses (JCPyV, BKPyV, MCPyV, TSPyV) have been clearly associated with acute disease at the time of initial infection. Rather, immunosuppression is a critical co-factor that is coupled to the ability of polyomaviruses to persist throughout life or to integrate into the genome, as in the case of MCPyV, ultimately leading to disease. Our study demonstrates that a large segment of the general population has been infected by STLPyV and might harbor persistent STLPyV infection, assuming the persistence paradigms of JCPyV and BKPyV hold true for STLPyV. A recent report identified STLPyV DNA in a skin wart specimen from an adult with primary immunodeficiency (*11*). Thus, it is critical to determine whether human diseases exist that are caused by STLPyV, especially in immunocompromised persons.

Technical Appendix Materials and methods.
