# Supplementary material for: Common Exposure to STL Polyomavirus During Childhood
Source: Emerg Infect Dis. 2014 Sep;20(9):1559–61. doi: 10.3201/eid2009.140561 (PMC4178396; doi:10.3201/eid2009.140561)
Supplement: Technical Appendix — Materials and methods. [file 14-0561-Techapp-s1.pdf]

Article DOI: http://dx.doi.org/10.3201/eid2009.140561

Common Exposure to STL Polyomavirus During Childhood

Technical Appendix

Materials and Methods

Clinical Specimens

A total of 500 serum samples (Denver, CO, USA) were obtained from healthy adult blood donors (May and June 2007) from Bonfils Blood Center (Denver), and serum samples from children were obtained from hospital blood draws at The Children's Hospital (Denver) with the exclusions previously described in (*1*). The protocols were approved by the Colorado Multiple Institutional Review Board as previously described (*1*). A total of 419 serum samples (Saint Louis, MO, USA) were obtained from a panel of available serum specimens collected from the clinical laboratories at Barnes-Jewish Hospital (Saint Louis) and St. Louis Children’s Hospital (Saint Louis) (November 2007–October 2008) as previously described (*2*), using protocols approved by the Human Research Protection Office of Washington University in St. Louis, School of Medicine. Samples were de-identified and analyzed anonymously.

Expression and Purification of Recombinant STL polyomavirus Glutathione S-Transferase–Tagged VP1 Fusion Protein

The *vp1* gene of STL polyomavirus (STLPyV) strain WD972 (GenBank accession no. JX463184) was synthesized (GenScript, Piscataway, NJ, USA) for *Escherichia coli* codon optimization and cloned into pGEX4T3 vector by using BamHI and XhoI restriction sites. N-terminal glutathione S-transferase (GST)–tagged STLPyV VP1 proteins were expressed in *E. coli* and purified by affinity chromatography as previously described (*1*). STLPyV VP1 capsomeres were prepared by on column cleavage of glutathione-sepharose bound GST VP1 by using thrombin. For electron microscopy, capsomeres were adsorbed onto copper grids and negatively stained with uranyl acetate. Images were captured with a 1Kx1K Gatan Bioscan digital camera on a Philips CM10 electron microscope operating at 80kV.

Capture ELISA

The ELISA was performed as previously described (*1*). In brief, Polysorp 96-well plates (Nunc, Naperville, IL, USA) were coated with casein-glutathione conjugate overnight at 4°C, after which GST-VP1 (100 ng/well) was bound for 1 h at 4°C. Serum samples were diluted 1:50 in block buffer (5% nonfat dry milk powder; 0.05% Tween 20 in phosphate buffered saline [PBS], pH 7.4) and incubated with the immobilized STLPyV GST-VP1 antigen for 1 h at room temperature, and then washed 3 times with 0.05% Tween 20 in PBS. IgG was detected by using an horseradish peroxidase (HRP)-conjugated secondary antibody (Promega, Madison, WI, USA), and developed with Ultra-tetramethylbenzidine substrate (Thermo Scientific, Waltham, MA, USA). Serum specimens were tested in triplicate. ELISAs for the Denver specimens were performed at University of Colorado Boulder, and ELISAs for the Saint Louis specimens were performed at Washington University. Twenty-eight random serum specimens were tested in both laboratories in a blinded fashion, and ELISA results were concordant in terms of both seropositivity and general intensity trends between both laboratories.

For the competition ELISAs, STLPyV VP1 (3.0 μg/mL) or MWPyV VP1 (manuscript in preparation) (3.0 μg/mL) capsomeres were pre-incubated with 24 serum samples arbitrarily selected across the age distribution (diluted 1:50) for 1 h at 4°C before adding to the immobilized STLPyV GST-VP1 antigen.

ELISA Cutoff Determination

Because ELISAs for the Denver and Saint Louis specimens were performed in separate laboratories, an independent cutoff value was determined for each site. Ranked net absorbance values was plotted and fit to a third-degree polynomial regression model (R2>0.99). The cutoff value was determined by the inflection point, corresponding to absorbance values of 0.276 (Denver) and 0.239 (Saint Louis).

References

1. Kean JM, Rao S, Wang M, Garcea RL. Seroepidemiology of human polyomaviruses. PLoS Pathog. 2009;5:e1000363. PubMed http://dx.doi.org/10.1371/journal.ppat.1000363

2. Nguyen NL, Le BM, Wang D. Serologic evidence of frequent human infection with WU and KI polyomaviruses. Emerg Infect Dis. 2009;15:1199–205. PubMed http://dx.doi.org/10.3201/eid1508.090270
